# A Case Report of Acute Diverticulitis in “Pseudodiverticulosis” after Hemorpex System® Procedure

**DOI:** 10.1155/2016/3298048

**Published:** 2016-11-15

**Authors:** Tommaso Testa, Federico Costanzo

**Affiliations:** Surgical Clinic Unit II, Department of Surgical Sciences and Integrated Diagnostics (DISC), University of Genoa, Genova, Italy

## Abstract

*Introduction*. In the last years many mini-invasive approaches were developed in order to reduce postoperative pain and complication after haemorrhoid surgery: one of these alternatives is represented by Hemorpex System, a relatively young technique that combines transanal dearterialization with mucopexy through a dedicated proctoscope.* Case Presentation*. A 78-year-old male patient was admitted to the Emergency Department for acute urinary retention and elevated temperature. Hemorpex procedure was performed 4 years before. Clinical, endoscopic, and radiological findings demonstrated the presence of multiple diverticula-like structures fulfilled by purulent fluid and a deep alteration of the normal anatomy of the rectum. He was treated following the standard protocol of acute diverticulitis and full recovery from symptoms was achieved.* Discussion*. Hemorpex System is a young technique, and nowadays-available studies lack long-term follow-up data. Anatomical changes induced by the procedure are consistent and definitive. Our patient luckily demonstrated a prompt response to conservative treatment, but it must be taken into account that, in case of medical treatment failure, surgical approach would be necessary and the actual patient anatomical changes could lead the surgeon to unavoidable threatening maneuvers.

## 1. Introduction

Since haemorrhoidal disease represents one of the most relevant proctological conditions in Western Countries, many efforts have been made during the last years in order to guarantee a less invasive treatment and an optimized management of the postoperative pain, bleeding, and recurrence [1]. Conventional approaches to haemorrhoids with various grade of prolapse typically include invasive surgery, such as haemorrhoidectomy (Milligan-Morgan and Ferguson's procedures) and stapled haemorrhoidopexy (Longo procedure). The introduction of ultrasound (Harmonic®, Ace®) and radiofrequency scalpel (LigaSure®) in Milligan-Morgan haemorrhoidectomy substantially reduced postoperative pain and accelerated healing [2, 3]; on the other hand the final cost of the procedure raised significantly. Supporters of Longo's haemorrhoidopexy claimed that this procedure would have carried advantages in terms of postoperative pain control and fast healing [4]. Nevertheless meta-analysis demonstrated that no significant differences are noticed between the two techniques considering pain level [5] and that recurrence rate is higher in those patient affected by grade IV haemorrhoids who underwent Longo procedure rather than haemorrhoidectomy, while no difference in recurrence was found in patient affected by grade II/III haemorrhoids [6]. The high cost of haemorrhoidopexy equipment could be justified by the prompt reintroduction of the patient to the work force [4] that is demonstrated to be slower in patients to whom haemorrhoidectomy was performed. Complications involve postoperative thrombosis, urinary retention, anal stenosis, abscess, and anal fistula formation as well as sphincter damages with resulting incontinence disturbs: no statistical differences were reported for each complication between haemorrhoidectomy and haemorrhoidopexy [4], even though rare but severe complications are described after Longo procedure (hemoperitoneum [7], acute rectal obstruction [8], bacteraemia [9], persistent postoperative pain, and lethal sepsis [10]).

Conservative alternatives [11] such as procedures of dearterialization [12] were developed with the aim of reaching a mini-invasive technique standard, feasible in an outpatient setting: Morinaga described the first arterial ligation in 1995, and since then the introduction of new dedicated instruments brought to significant results. Modern proctoscopes allow ligation of the terminal branches of the haemorrhoidal artery: the reduction of blood flow leads to the shrinkage of haemorrhoidal cushion and subsequent improvement of the symptoms [13]. A transanal Doppler probe allows a precise identification of the arterial branches that need suturing, even though Gupta et al. [11] recently suggested that no substantial contributions are carried by Doppler guidance. Dearterialization has the proven advantages of a quick recovery time, a low postoperative pain, and the restoration of the physiology of haemorrhoids. Giordano et al. [13] reviewed a total of 1996 patients, reporting the complication rate of dearterialization: postoperative pain was experienced by 18.5% of patients, followed by residual protrusion, bleeding, fever, and thrombosed haemorrhoid and complications such as anal fissures, urinary retention, incontinence, fistulas, proctitis, and stool retention in less than 1% of patients. In a 12-month follow-up study [14], recurrence rates were reported considering preoperative haemorrhoidal degree: the relapse rate was 4.8% for third-degree and 26.7% for fourth-degree haemorrhoids: this brought to the conclusion that dearterialization represents a valid alternative for grades II and III haemorrhoids but not for grade IV.

Hemorpex System (first studies started in 2003) combines transanal dearterialization with mucopexy through a dedicated proctoscope and seems to offer a valid alternative to traditional haemorrhoidectomy in grades II and III haemorrhoidal disease [15, 16]. The device cost is approximatively 300€ in Italy: this is set between Longo haemorrhoidopexy (mean 500€) and Milligan-Morgan haemorrhoidectomy (approximatively 250€). According to Tagliabue et al. [17] and Iachino et al. [15] postoperative pain was well controlled. No acute postoperative bleeding was reported by Tagliabue et al., but 3.4% of patients experimented severe bleeding within 15 days after surgery and needed surgical revision; these data are in contrast with Iachino study, in which no cases of acute bleeding were recorded and 13.5% of patients reported only mild blood loss when defecating within 2 weeks after surgery. At a 30-day follow-up, anal canal discomfort was the most represented complication (30.3%), followed by tenesmus and perianal ecchymosis; fistula, abscess, haematospermia and pseudopolyps were described in less than 1% of the patients. Recurrences were assessed in about 1.6% of grade II haemorrhoids patients, 3.4% in grade III, and 37.5% in grade IV. The learning curve of this technique is relatively short if compared to other procedures, complication rate (including pain, bleeding, and recurrence for grades II and III) is low, and the full recovery of the patient is fast.

## 2. Case Presentation

**Figure 1:**
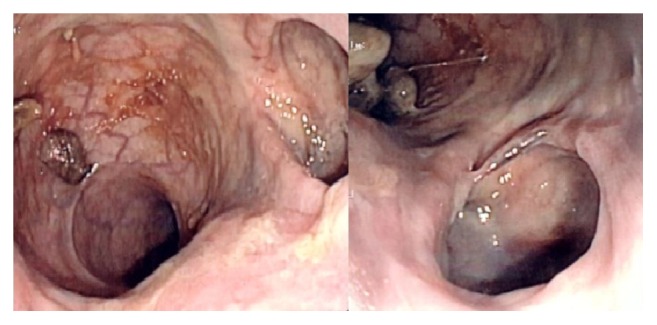
Endoscopic view of pseudodiverticula of the rectum.

**Figure 2:**
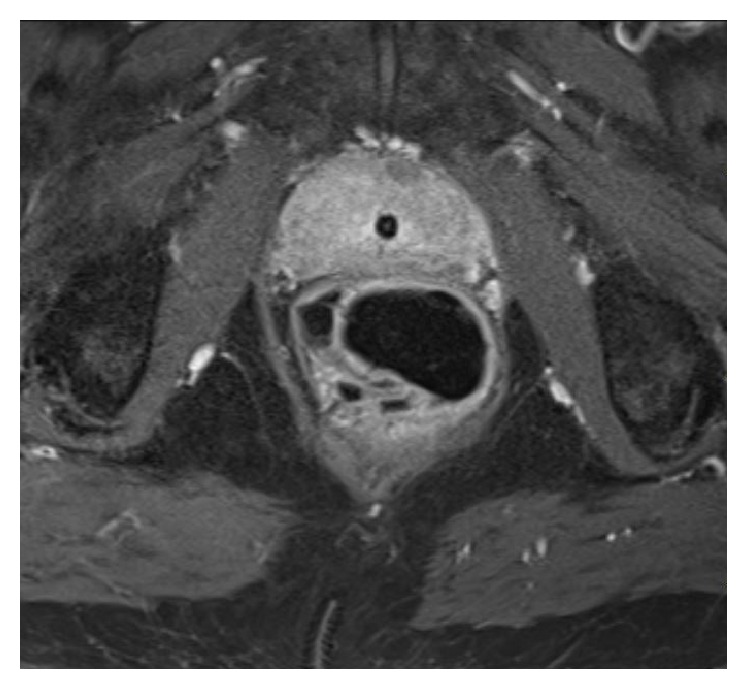
MRI showing anatomical changes of the rectum.

A 78-year-old man was admitted to the Emergency Department for acute urinary retention and elevated temperature (38.5°C). Diagnosis at admission was acute prostatitis: urinary catheter was placed and broad-spectrum antibiotics therapy was administered. Due to symptoms persistence, the patient underwent proctological evaluation: digital rectal examination was extremely painful and a thickened area of the anterior wall of the anal canal extending upward (4-5 cm) from the pectinate line was detected. Anoscopy was also performed, but the presence of abundant purulent discharge did not allow a complete evaluation of the distal portion of the rectum. An endoscopic examination through colonoscopy was requested (Figure 1): it revealed the presence of multiple diverticula-like structures fulfilled by purulent fluid. An MRI was finally performed (Figure 2): distal rectum presented multiple outpouching, air-filled hollows (the major measuring 44 mm and the minor measuring 20 mm, right anteriorly to the rectum). The patient previously underwent HPS procedure at least 4 years before: the postoperative period was uneventful; the patient remained asymptomatic until that moment; first clinical check after surgery was normal. He was treated following the standard protocol of acute diverticulitis, that is, i.v. ampicillin and sulbactam (2 g + 1 g × 3/die) and metronidazole (500 mg × 2/die), no feeding, and i.v. fluid support for 7 days, followed by oral metronidazole 500 mg × 2/day. Urinary catheter was removed after two weeks, antibiotics were stopped, and full recovery from symptoms was achieved as well as normalization of laboratory test values.

## 3. Discussion

Prevalence of haemorrhoidal disease is around 5% in general population of Western Countries, but not more than 10% of whom does effectively need surgery. Surgical approach to haemorrhoids is still a debated matter. A constant effort is made in order to find out a “passe-partout” technique that could fit properly each possible situation: this attempt, even though practical, could lead to mistreating errors. Results, on the contrary, support the idea of a “patient-tailored” approach instead of the “technique-tailored” or even worse “surgeon-tailored” ones: this requires a wide-experienced operator but ensures to the patient the optimal treatment for his own specific condition. Milligan-Morgan procedure is one of the most popular techniques adopted all over the world because of good long-term results, even though (mild) postoperative pain is accused and healing period is not so quick. Significant progresses have been achieved in terms of shortening of hospitalization, better pain control, and fast healing through the introduction of new instruments, such as radiofrequencies and ultrasounds scalpels. The cultural revolution introduced by Longo, despite an initial success due to the reduction of postoperative pain, did not succeed in becoming the gold standard in the treatment of grades II and III haemorrhoids; a reason could be found into the high percentage of relapses and the seriousness of the possible complications.

**Figure 3:**
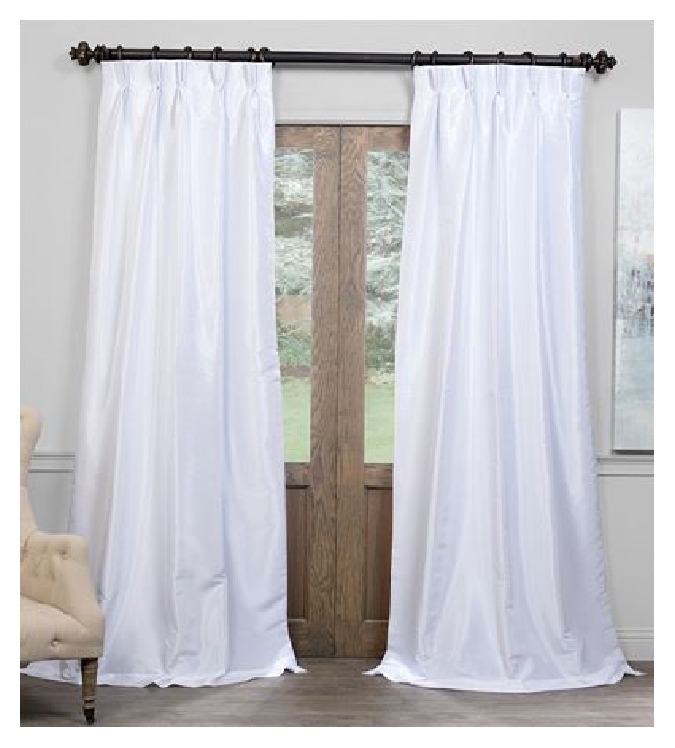
Preoperative mucosal prolapse.

**Figure 4:**
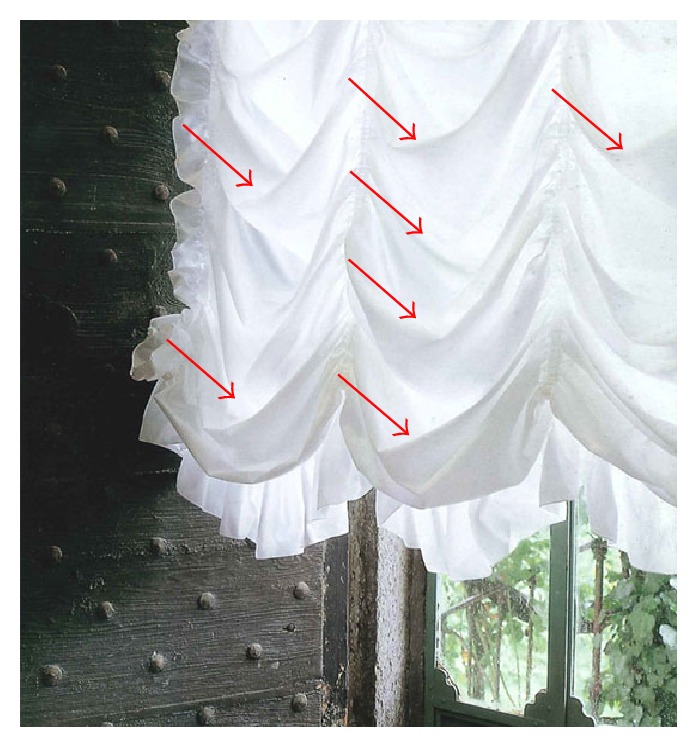
Postoperative outpouching pseudodiverticular formation.

Techniques of dearterializing mucopexy are getting popular because of short learning curve, low grade of acute/short term complications, and fast recovery after procedure. The therapeutic aim of the procedure is based on the manual suturing of the mucosal prolapse, without the risk linked to a standard prolapsectomy. According to many studies, results are encouraging, and even though this technique requires general or locoregional anaesthesia (not convenient for an outpatients setting) both the percentage of complications and the cost of the procedures (due to the specific proctoscope) are affordable. Despite this, it must be underlined that most of the studies nowadays available come from the same country (Italy), patient cohorts are poor (or astonishingly enormous for medium population cities), and follow-ups weak with a high rate of postoperative drop-outs and no systematic controls on data are applied (a study was even withdrawn because of declaration of false data [18]): therefore no definitive evaluation could be established for long-term outcomes since this technique is relatively young. The case we describe of “pseudodiverticulitis of the rectum” is the first case of a late-onset complication after HPS reported in literature. Our explanation of the clinical findings of the patient could be well represented by the effect of a draped curtain (Figures 3 and 4), where each plication of the cloth resembles a diverticulum-like formation (Figure 4, red arrows) susceptible of stool collection and subsequent infection.

This appears clearly through endoscopic examination, but MRI findings are also surprising: from an anatomic point of view, this “iatrogenic pseudodiverticulosis” of the rectum appears as a chronic stabilized condition with definitive anatomic changes. Of course, it is not possible to state that the anatomical changes revealed are due to a badly performed technique rather than the normal consequence of a well performed one. We reported a prompt clinical response to antibiotics and the avoidance of enteral feeding in this first episode of acute diverticulitis. One additional approach we adopted, of course not standardized due to the lack of other similar cases, was the prescription of a monthly Rifaximin therapy, decision based on the clinical similarity to a typical diverticulitis of the colon. This treatment allowed the total remission of the acute episode through conservative approach, with no need of surgical revision, which would have implied both decisional and technical difficulties (resection of the involved rectal segment, performance of a coloanal anastomosis, and protective stoma). With the hope of no-recurrence for our patient, we still cannot foresee whether an eventual second inflammatory episode would be responsive to conservative treatment: if not, the only rescue treatment would contemplate the above surgical approaches. Is it worth taking the risk?
